# Thermal servo-controlled systems in the management of VLBW infants at birth: A systematic review

**DOI:** 10.3389/fped.2022.893431

**Published:** 2022-08-01

**Authors:** Orietta Ercolino, Erica Baccin, Fiorenza Alfier, Paolo Ernesto Villani, Daniele Trevisanuto, Francesco Cavallin

**Affiliations:** ^1^Department of Woman's and Child's Health, University Hospital of Padua, Padua, Italy; ^2^Department of Woman's and Child's Health, Fondazione Poliambulanza, Istituto Ospedaliero, Brescia, Italy; ^3^Independent Statistician, Solagna, Italy

**Keywords:** preterm infants, servo-controlled system, temperature, delivery room, review—systematic

## Abstract

**Background:**

Thermal management of the newborn at birth remains an actual challenge. This systematic review aimed to summarize current evidence on the use of thermal servo-controlled systems during stabilization of preterm and VLBW infants immediately at birth.

**Methods:**

A comprehensive search was conducted including MEDLINE/Pubmed, EMBASE, SCOPUS, clinicaltrials.gov, and the Cochrane Database through December 2021. PRISMA guidelines were followed. Risk of bias was appraised using Cochrane RoB2 and Risk Of Bias In Non-Randomized Studies of Interventions (ROBIN-I) tools, and certainty of evidence using GRADE framework.

**Results:**

One randomized controlled trial and one observational study were included. Some aspects precluded the feasibility of a meaningful meta-analysis; hence, a qualitative review was conducted. Risk of bias was low in the trial and serious in the observational study. In the trial, the servo-controlled system did not affect normothermia (36.5–37.5°C) but was associated with increased mild hypothermia (from 22.2 to 32.9%). In the observational study, normothermia (36–38°C) increased after the introduction of the servo-controlled system and the extension to larger VLBW infants.

**Conclusion:**

Overall, this review found very limited information on the use of thermal servo-controlled systems during stabilization of preterm and VLBW infants immediately at birth. Further research is needed to investigate the opportunity of including such approach in the neonatal thermal management in delivery room.

**Registration:**

PROSPERO (CRD42022309323).

## Introduction

The transition between intrauterine and extrauterine life depends on anatomic and physiologic changes that occur at birth. Although most newborns do not require any type of assistance to make this transition successfully, 5–10% require additional interventions such as resuscitation in the delivery room (1). Preterm infants are more likely to need resuscitation and experience associated complications, especially very low birth weight (VLBW) and extremely low birth weight (ELBW) infants (1, 2).

In the delivery room, preventing thermal losses of the newborn is crucial, because hypothermia in the immediate postnatal period is associated with increased morbidity (i.e., respiratory distress syndrome, metabolic disorders, intraventricular hemorrhage, late-onset sepsis) and mortality (1, 3–7). In particular, preterm and VLBW babies are at very high risk of rapid heat loss because of their major body surface area-to-mass ratio, relative thin skin and poor subcutaneous fat tissue (8). On the other hand, the U-shape association between neonatal temperature and unfavorable outcome suggests that hyperthermia should be avoided as well (9, 10).

The goal of thermal care is maintaining body temperature in the interval 36.5–37.5°C (1, 3). Various strategies have been implemented in the clinical practice to minimize heat loss in preterm infants, including occlusive wrapping, exothermic mattresses, polyethylene or wool caps, warmed humidified gasses, increasing delivery room temperature to 26°C, and radiant warmers (11).

These interventions have been used as a single or in combination as a bundle, with different degrees of success (1). A recent Cochrane systematic review showed that external heat sources may reduce hypothermia risk in preterm and/or low birth weight infants, but also suggested caution to avoid iatrogenic hyperthermia (12). A thermal servo-controlled system uses the thermal feedback by the patient to optimize the thermal output of the infant warmer reducing both hypothermia and hyperthermia risks. The thermal servo-controlled systems are regularly employed in neonatal intensive care unit (NICU) for thermal management, but they can provide some advantages when used in the delivery ward. This systematic review aimed to summarize current evidence on the use of thermal servo-controlled systems during stabilization of preterm and VLBW infants immediately at birth.

## Materials and methods

### Study design

This is a systematic review of comparative studies assessing thermal management with servo-controlled warmers vs. other thermal interventions in preterm and VLBW infants. The review was conducted following the Preferred Reporting Items for Systematic Reviews and Meta-Analyses (PRISMA) guidelines (13). The protocol was registered in PROSPERO (CRD42022309323).

### Search strategy

We systematically searched MEDLINE/PubMed, EMBASE, SCOPUS, Cochrane Central Register of Controlled Trials, and Clinicaltrials.gov. The search was implemented without language restrictions from database inception until February 8, 2022. In MEDLINE/PubMed, the search strategy was: (servo control^*^) AND (neonat^*^) AND (temp^*^) NOT (cooling). This search strategy was tailored to the other electronic sources. We also hand-searched the reference list of retrieved papers to detect further articles of interest. Two investigators (EB, FA) independently screened titles and abstracts, and obtained the full text of all potentially eligible articles. Discrepancies at any stage of the process were settled by consensus with the review team.

### Inclusion criteria

Study design: Randomized controlled trials, non-randomized controlled trials, interrupted time series, before-and-after implementation studies.

Population: Preterm infants (<37 weeks' gestational age) and/or VLBW infants (birth weight < 1,500 g).

Intervention: Use of servo-controlled system in delivery room.

Comparator: Any other thermal interventions without a servo-controlled system.

Outcomes: Neonatal temperature at NICU admission, morbidity (intraventricular hemorrhage, respiratory distress syndrome, late onset sepsis, bronchopulmonary dysplasia), adverse events, mortality before hospital discharge.

Time: From database inception until February 8, 2022.

Conference abstracts and trial protocols were excluded. Studies not including human subjects were excluded.

No language restrictions were applied if there was an English abstract.

### Data collection

Two investigators (EB, FA) independently retrieved relevant data from selected studies, including study characteristics (study design, year of publication, country), study population (number, and age of enrolled patients), type of intervention, and outcomes measures. A third investigator (OE) supervised data extraction. Study authors were contacted, when appropriate, to request additional unpublished data.

### Assessment of risk of bias and certainty of evidence

Two investigators (EB, FA) independently evaluated the included studies. The Risk Of Bias In Non-Randomized Studies of Interventions (ROBINS-I) was used for the non-randomized study comparing different interventions (14). The Cochrane revised tool to assess risk of bias in randomized controlled trials (RoB 2.0) was used for the randomized controlled trial (15). Any discrepancy was settled by consensus with the review team. The certainty of evidence from the randomized controlled trial was assessed by using the GRADE framework (16).

### Data synthesis

In the review protocol, we anticipated the intention of performing a formal data analysis with meta-analytical techniques. Unfortunately, the search yielded only two eligible studies with different design, thus precluding the feasibility of a meaningful meta-analysis. Hence, a qualitative narrative of these studies was conducted.

## Results

### Search results

**Figure 1:**
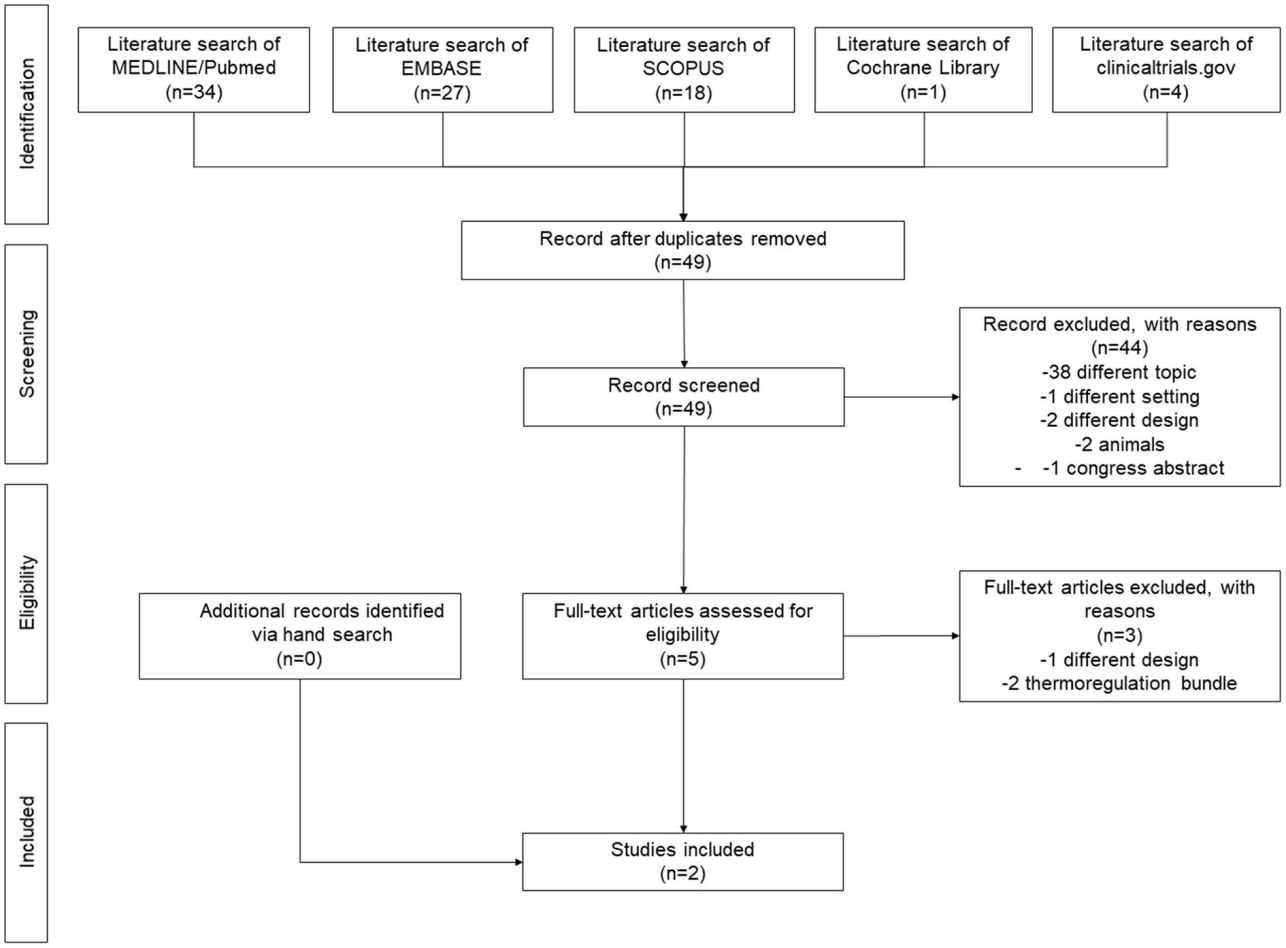
PRISMA flow diagram.

The search strategy identified 49 non-duplicated records. After excluding 44 articles based on title/abstract, 5 articles were retrieved for full-text review (17–21). Of these, one was excluded due to different setting since it was implemented in the neonatal intensive care unit (17). Two studies were excluded because the specific impact of the servo-controlled system could not be assessed as it was part of a thermoregulation bundle including multiple interventions (i.e., heated mattress, plastic wrapping, staff education) (18, 19). No further articles were identified *via* hand search; thus, a total of two studies (20, 21) were included in the qualitative narrative (Figure 1).

### Study and patient characteristics

The review included an observational study (20) and a randomized controlled trial (21) (Supplementary Table 1). Pinheiro et al. (20) reported an observational study comparing admission temperature before and after the implementation of a thermoregulation bundle, which included servo-controlled, battery-powered radiant warmers for stabilization and transfer starting from the 7th month of intervention. The study included 641 very low birth weight infants (<1,500 g) born in a US center in 2007–2012. Cavallin et al. (21) reported a multicenter, unblinded, randomized clinical trial comparing thermal management with and without the use of a servo-controlled system immediately after birth. The trial enrolled 450 preterm infants (estimated birth weight <1,500 g and/or gestational age <30 weeks) in 15 Italian tertiary hospitals between March 2019 and February 2020.

Both studies (20, 21) set temperature probe at 37°C and assessed thermal outcome measures (proportion of normothermia, hypothermia and hyperthermia at NICU admission) (20, 21) (Table 1). The trial also reported morbidity, mortality and occurrence of adverse events (21).

**Table 1:** Temperature at NICU admission.

**Study**	**Group**	**N participants**		**Neonatal temperature at NICU admissions**		
			<36°C	36–36.5°C	36.5–37.5°C	37.5–38°C	>38°C
Pinheiro et al. (20)	Before thermoregulation bundle	164	34.1%	64.6%			1.2%
	Thermoregulation bundle	62	17.7%	79.0%			3.2%
	Thermoregulation bundle +servo-controlled, battery-powered radiant warmers	440	4.6%	93.5%			1.9%
Cavallin et al. (21)	Thermal management with a servo-controlled system	225	26.7%	22.2%	39.6%	Not available	0.0%
	Thermal management without a servo-controlled system	225	27.6%	32.9%	42.2%	Not available	2.7%

### Risk of bias and certainty of evidence

The risk of bias is reported in Supplementary Table 2. According to ROBINS-I tool, the observational study (20) was at serious risk of bias for selection of participants (the intervention was later extended to neonates >28 weeks' gestation, who are less likely to experience hypothermia) and was judged to be at overall serious risk of bias. According to RoB2 tool, the trial (21) was at low risk of bias for all domains and was judged to be at overall low risk of bias. In addition, the certainty of evidence was high for the trial (21) (Supplementary Table 3).

### Qualitative narrative

Both studies reported thermal outcomes at NICU admission. The observational study (20) reported an increase from 79 to 93.5% of the proportion of infants in the thermal range of 36–38°C after the introduction of the servo-controlled system and the extension to infants >28 weeks' gestation. Both hypothermia (<36°C) and hyperthermia (>38°C) rates decreased after the implementation (Table 1). In the trial (21), the servo-controlled system did not significantly influence the rates of normothermia (36.5–37.5°C), moderate hypothermia (<36°C) and hyperthermia (>38°C), but it was associated with increased mild hypothermia (from 22.2 to 32.9%).

In addition, the trial did not report any significant differences between thermal with vs. without servo-controlled system in terms of morbidity (intraventricular hemorrhage, respiratory distress syndrome, late onset sepsis, bronchopulmonary dysplasia), adverse events (neonatal temperature <35 or >39°C) and in-hospital mortality (Supplementary Table 4).

## Discussion

This systematic review found very limited information on the use of thermal servo-controlled systems during stabilization of preterm and VLBW infants immediately at birth. Only two studies satisfied the inclusion criteria and could be considered in the review. A multicenter randomized controlled trial (21) showed that the servo-controlled system did not improve normothermia at NICU admission in very low birthweight infants. Differently, an observational study (20) suggested that using a thermal servo-controlled system may improve normothermia at NICU admission in preterm infants. Of note, caution is required in the interpretation of these findings because the introduction of the servo-controlled system overlapped with the extension of the treatment to larger VLBW infants (20) who are less prone to hypothermia (19). In addition, the introduction of the servo-controlled system within the implementation of a thermoregulation bundle over time implied an effect of the quality improvement curve on the final results. We believe that such aspect (alongside the different study design and participants) may contribute to explain the large difference in the proportion of infants within 36–38°C between the trial and the observational study (73.3 vs. 93.5%).

In the trial, the servo-controlled system prevented hyperthermia but increased mild hypothermia at NICU admission (21). The authors speculated that such increase might be imputed to the patient-driven thermal management, which might need longer time to achieve normothermia compared with an external-driven thermal management (maximum thermal output set by the healthcare provider). This might be overcome by increasing the temperature set in the servo-controlled system (i.e., 37.5°C), but the patient could be exposed to higher risk of hyperthermia.

In addition, the trial did not find any significant differences between thermal management with vs. without servo-controlled system in terms of morbidity, adverse events and in-hospital mortality (21). Such information was not reported in the observational study (20).

The literature offered some information on the implementation of thermoregulation bundles including the servo-controlled system among other interventions, such as staff education and thermal-oriented equipment (i.e., heated mattress, plastic wrapping, staff) (18, 19). Young et al. described a quality improvement initiative for very preterm infants based on a neonatal stabilization team of four members with specific tasks (inspired to the Formula 1 motor racing pit stop), in which the servo-controlled system was activated with or without a Transwarmer if the neonatal temperature remained below 36.5°C (18). Bhatt et al. (19) implemented a thermoregulation bundle for extremely low birthweight infants with 17 elements covering perinatal aspects, staff education and debriefing, dedicated delivery site, several warming devices (including the servo-controlled system) and continuous recording of the temperature. Unfortunately, the inclusion of the servo-controlled system in a thermoregulation bundle did not allow the appraisal of its separate contribution, hence such studies could not be included in this review.

To our knowledge, there are no published economic or cost-effectiveness data on the use of the servo-controlled system in the delivery ward, but it is likely that the costs are higher than a manual set infant warmer. Although the magnitude of the difference is unknown, this may result in health inequities in low-resource settings. Of course, this consideration is subordinate to the future assessment of the effectiveness of the servo-controlled system on important clinical outcomes.

The strengths of this systematic review include the pre-specified published protocol, the literature search performed by two authors independently and the adherence to PRISMA guidelines. However, the reader should be aware of the limitations of the review. In fact, the different study design and interventions of included studies precluded the pooling of the results, thus limiting the summary of the findings to a qualitative narrative. Similar aspects also precluded a reasonable assessment of the certainty of evidence, hence preventing the authors from drawing strong conclusions.

## Conclusions

This systematic review found very limited information on the use of thermal servo-controlled systems during stabilization of preterm and VLBW infants immediately at birth. Further studies comparing thermal management of preterm infants at birth with vs. without a servo-controlled system are required to provide information to health care providers and stake holders about the opportunity of including such approach in the neonatal thermal management in delivery room.

## Author contributions

OE prepared the protocol, screened studies, participated in the analysis, and prepared the first draft of the manuscript. EB and FA screened studies, abstracted data, completed risk-of-bias, participated in the analysis, and prepared the first draft of the manuscript. PV was involved in reviewing the protocol, reviewing the analysis, and writing and editing the manuscript. DT conceptualized the study, prepared the protocol, completed the analysis, and contributed to writing and editing the manuscript. FC prepared the protocol, performed the literature search, supervised the analysis, and contributed to writing and editing the manuscript. All authors approved the final manuscript as submitted and agree to be accountable for all aspects of the work.

## Conflict of interest

The authors declare that the research was conducted in the absence of any commercial or financial relationships that could be construed as a potential conflict of interest.

## Publisher's note

All claims expressed in this article are solely those of the authors and do not necessarily represent those of their affiliated organizations, or those of the publisher, the editors and the reviewers. Any product that may be evaluated in this article, or claim that may be made by its manufacturer, is not guaranteed or endorsed by the publisher.
