# Supplementary material for: Thermal servo-controlled systems in the management of VLBW infants at birth: A systematic review
Source: Front Pediatr. 2022 Aug 1;10:893431. doi: 10.3389/fped.2022.893431 (PMC9377414; doi:10.3389/fped.2022.893431)
Supplement: Supplementary file 1 [file Table_1.DOCX]

Supplementary Table 1 Characteristics of included studies

| Study | Country | Study design | Enrolled participants | Participants | Intervention | Control | Outcome measure |
| --- | --- | --- | --- | --- | --- | --- | --- |
| Pinhero et al. (2014) | United States | Observational study | 641 | Birth weight <1500 grams | -Thermoregulation bundle (consistent head and torso wrapping with plastic, warmed blankets, a closed stabilization room)  -After 6 months, thermoregulation bundle +servo-controlled, battery-powered radiant warmers for stabilization and transfer | Radiant warmers  (without battery packs) for stabilization and transfer, inconsistent  wrapping and use of prewarmed blanket,inconsistent drying, unregulated temperature measurements | Neonatal temperature at NICU admission |
| Cavallin et al. (2021) | Italy | Randomized controlled trial | 450 | Estimated birth weight <1500 grams and/or gestational age <30 weeks | Thermal management with a servo-controlled system | Thermal management without a servo-controlled system | Neonatal temperature at NICU admission; morbidity ^a^;  adverse events ^b^; in-hospital mortality |

^a^Morbidity included: intraventricular hemorrhage, respiratory distress syndrome, late onset sepsis, bronchopulmonary dysplasia. ^b^Adverse events included severe hypothermia (<35°C) or hyperthermia (>39°C).

Supplementary Table 2. Risk of bias of included studies

| Study | Tool | | | | | | | | | | | | |
| --- | --- | --- | --- | --- | --- | --- | --- | --- | --- | --- | --- | --- | --- |
|  | ROBINS-I (observational study) | | | | | | | | | | | | |
|  | Confouding | Selection of participants | | Classification of intervention | | Deviations from intended interventions | | Missing outcome data | Measurement of outcomes | | Selection of reported results | | Overall bias |
| Pinhero et al. (2014) | Low | Serious | | Low | | Low | | Low | Low | | Low | | Serious |
|  | RoB2 (randomized controlled trial) | | | | | | | | | | | | |
|  | Randomization process | | Deviations from the intended interventions | | Missing outcome data | | Measurement of the outcome | | | Selection of the reported result | | Overall bias | |
| Cavallin et al. (2021) | Low | | Low | | Low | | Low | | | Low | | Low | |

Supplementary Table 3. GRADE assessment for the randomized controlled trial included in the review

| **Certainty assessment** | | | | | | | **№ of patients** | | **Effect** | | **Certainty** | **Importance** |
| --- | --- | --- | --- | --- | --- | --- | --- | --- | --- | --- | --- | --- |
| **№ of studies** | **Study design** | **Risk of bias** | **Inconsistency** | **Indirectness** | **Imprecision** | **Other considerations** | **Servo-controlled system** | **No servo-controlled system** | **Relative (95% CI)** | **Absolute (95% CI)** |  |  |
| **Normothermia** | | | | | | | | | | | | |
| 1 | randomised trials | not serious | not serious | not serious | not serious | none | 89/225 (39.6%) | 95/225 (42.2%) | **RR 0.94** (0.75 to 1.17) | **25 fewer per 1.000** (from 106 fewer to 72 more) | ⨁⨁⨁⨁ High | IMPORTANT |
| **Mild hypothermia** | | | | | | | | | | | | |
| 1 | randomised trials | not serious | not serious | not serious | not serious | none | 74/225 (32.9%) | 50/225 (22.2%) | **RR 1.48** (1.09 to 2.01) | **107 more per 1.000** (from 20 more to 224 more) | ⨁⨁⨁⨁ High | IMPORTANT |
| **Moderate to severe hypothermia** | | | | | | | | | | | | |
| 1 | randomised trials | not serious | not serious | not serious | not serious | none | 60/225 (26.7%) | 62/225 (27.6%) | **RR 0.83** (0.71 to 1.31) | **47 fewer per 1.000** (from 80 fewer to 85 more) | ⨁⨁⨁⨁ High | IMPORTANT |
| **Hyperthermia (>38°C)** | | | | | | | | | | | | |
| 1 | randomised trials | not serious | not serious | not serious | not serious | none | 0/225 (0.0%) | 6/225 (2.7%) | **RR 0.08** (0.00 to 1.36) | **25 fewer per 1.000** (from -- to 10 more) | ⨁⨁⨁⨁ High | IMPORTANT |

**CI:** confidence interval; **RR:** risk ratio

Supplementary Table 4. Morbidity, adverse events, and in-hospital mortality

| Study | Group | N participants | Intraventricular hemorrhage (all grades) | Respiratory distress syndrome | Late onset sepsis | Bronchopulmonary dysplasia | Neonatal temperature <35°C | Neonatal temperature >39°C | In-hospital mortality |
| --- | --- | --- | --- | --- | --- | --- | --- | --- | --- |
| Cavallin et al. (2021) | Thermal management with a servo-controlled system | 225 | 30 (13.3%) | 179 (79.6%) | 39 (17.3%) | 46 (20.4%) | 6 (2.7%) | 0 (0.0%) | 16 (7.1%) |
|  | Thermal management without a servo-controlled system | 225 | 33 (14.7%) | 182 (80.9%) | 28 (12.4%) | 47 (20.9%) | 2 (0.9%) | 0 (0.0%) | 26 (11.6%) |
